# Neuropsychiatric Symptoms in an Inpatient Parkinson's Disease Sample

**DOI:** 10.1155/2014/420240

**Published:** 2014-03-12

**Authors:** Nicole C. R. McLaughlin, Irene Piryatinsky, Gary Epstein-Lubow, Louis Marino, Joseph H. Friedman

**Affiliations:** OCD Research Clinic, Butler Hospital, Alpert Medical School of Brown University, 345 Blackstone Boulevard, Providence, RI 02906, USA

## Abstract

*Background*. Neuropsychiatric symptoms are common in idiopathic Parkinson's disease (PD), and hospitalization for delirium, depression, psychosis, and anxiety is sometimes required. A minimal amount of data exists on these patients. *Methods*. Charts of all patients admitted to a psychiatric hospital between 2006 and 2009 with a diagnosis of PD were reviewed. Forty-three met entry criteria and were reviewed. Initial and discharge diagnoses, comorbid psychiatric diagnoses, length of stay, and living arrangements before and after hospitalization are described. *Results*. Consistent with previous research, this study showed evidence of comorbid psychiatric disorders within PD. *Conclusions*. The long-term goal of this area of study would be to reduce neuropsychiatric symptoms and improve quality of life in order to reduce inpatient hospital stays.

## 1. Introduction 

Idiopathic Parkinson's disease (PD) is a progressive neurodegenerative disorder classified as a movement disorder. Diagnostic criteria require defining motor features and the absence of atypical features, including dementia. Until recently, studies have focused on motor symptoms in PD, with minimal attention to common nonmotor symptoms often associated with the disease.

Although prevalence levels vary depending on methodology, neuropsychiatric symptoms are clearly prominent in PD. Depression affects 40–56% of individuals with PD [1–3], while apathy has been shown in 38.8–60% [1, 2]. Though not as frequently studied, anxiety affects 16.7–43% [2–6]. Impulse control disorders (ICDs) are found in 6–25% of the PD population taking dopamine agonists [7, 8]. Though few reports indicate the prevalence of substance abuse in PD, it has been found to be a risk factor for the development of ICDs with dopamine agonists [9]. Psychotic symptoms occur in 20–30% of patients who are receiving dopamine agonists [3, 10]. Nonmotor symptoms, including depression, anxiety, and REM sleep behavior disorder, may precede motor symptoms of PD [11]. Approximately 20–50% of individuals with PD have cognitive impairments, and the cumulative lifetime prevalence of dementia is 80% [3, 12–17]. Despite their prevalence, neuropsychiatric symptoms are underreported by neurologists and may go unrecognized by primary care physicians [18, 19].

Neuropsychiatric symptoms affect quality of life for both patients and caregivers. Low mood, depression, and hallucinations affect quality of life, even after controlling for motor symptoms [2]. Apathy, anxiety, and depression are all associated with quality of life [1, 20, 21], with depression as the largest predictor [18, 22]. Psychosis in PD is more stressful for caregivers than motor dysfunction and is the greatest risk factor for nursing home placement [23–25].

Neuropsychiatric symptoms can be severe enough to warrant inpatient psychiatric treatment. We carried out a retrospective chart-review study examining PD patients who required psychiatric hospitalization. To our knowledge, this is the first study examining inpatient psychiatric hospitalization in PD.

## 2. Methods

This study used a retrospective, chart-review design to examine characteristics of PD patients who required inpatient psychiatric hospitalization. Patients were hospitalized at Butler Hospital in Providence, Rhode Island, a private psychiatric facility with both inpatient and outpatient services. The hospital has adult treatment, substance abuse, adult intensive treatment, and geriatric units; data for this study was taken from any unit. All patients both diagnosed with PD and hospitalized for psychiatric reasons from 2006 to 2009 were included. The Butler Hospital Institutional Review Board approved this study. The study aimed to determine the following.

### 2.1. Initial and Discharge Diagnoses

The initial diagnosis was completed by a psychiatrist during hospital intake. All patients were followed by an attending psychiatrist throughout the hospital course; the attending psychiatrist assigned final diagnoses upon discharge.

### 2.2. Comorbid Psychiatric Diagnoses

Comorbid psychiatric conditions were recorded at the time of admission and during the hospital stay. Patient diagnoses are documented in standard clinical fashion. Upon admission, the patient (and family caregiver if available) is interviewed and historic records are reviewed to determine preliminary diagnoses (based upon DSM-IV criteria) for admission. During the course of inpatient treatment, diagnostic description is revised based on new data, such that the diagnoses at discharge represent the physician's assessment of the patient over time.

### 2.3. Length of Patient Stay

The length of stay was recorded from the date of initial assessment to the date of discharge for each patient's most recent hospital stay.

### 2.4. Living Arrangements before and after Hospitalization

Living arrangements prior to hospitalization and discharge arrangements were recorded. Primary categories of living arrangements included personal home, defined as a patient's own home (with possible assistance from a caregiver); home with formal assistance (outside agency); family or friend's home; assisted living facility; nursing home facility; and skilled nursing/medical facility.

## 3. Participants

Fifty-six inpatient charts were reviewed. Patients were included if they had a diagnosis of PD. Thirteen records were removed because it was unclear if the patient had idiopathic PD, as opposed to Parkinson's syndrome. Forty-three patients remained in the current sample. Seven of those patients had diagnoses of dementia with Lewy bodies. The sample ranged in age from 37 to 94 years, with a mean of 74.56 (SD 11.57). There were 27 female and 16 male patients.

Patients with comorbid diagnoses were included in more than one nominal category. Patients were categorized into 6 different groups based upon the DSM-IV [26], including (1) substance-related disorders; (2) schizophrenia and other psychotic disorders; (3) dementia, delirium, and amnestic and other cognitive disorders; (4) mood disorders; (5) anxiety disorders; and (6) adjustment disorders.

## 4. Results

### 4.1. Initial and Discharge Diagnoses

The most commonly diagnosed category upon intake was mood disorders, followed by dementia, delirium, and amnestic and other cognitive disorders (see Table 1). 27.9% of the sample had been diagnosed with dementia of the Alzheimer type, and 25.6% of the sample had been diagnosed with major depressive disorder (MDD). Fourteen percent of the sample had a substance-related disorder diagnosis. Two patients were diagnosed with alcohol abuse, one with alcohol dependence, one with anxiolytic dependence and opioid abuse, one with cannabis abuse, and one with barbiturate and alcohol dependence and cannabis abuse.

The most common diagnosis at discharge was MDD, in 25.6% of the sample (see Table 1). Five of the 12 patients' diagnoses of dementia of the Alzheimer type (DAT) were changed to DLB (1), MDD (1), delirium NOS (1), psychotic disorder NOS (1), and substance intoxication (1). At discharge, 16.3% of patients had diagnoses of a substance-related disorder, two with alcohol abuse, and one with alcohol dependence. One patient had barbiturate dependence, one had anxiolytic dependence, one had barbiturate and opioid dependence, and one had a diagnosis of substance intoxication. Fourteen patients in the sample showed no change in diagnosis from pre- to posthospitalization.

### 4.2. Length of Patient Stay

The average length of stay within the entire sample was 17.63 days (SD 18.19), with a range of 1–94 days. Qualitatively, patients with a substance-related disorder had the longest stays, and those with anxiety disorders diagnoses had the shortest stays, aside from one patient with an adjustment disorder diagnosis (see Table 1).

### 4.3. Living Arrangements before and after Hospitalization

#### 4.3.1. Prehospitalization

The largest number of patients (46.5%) came from their homes, either with or without family support (see Table 2). Almost 42% came from either an assisted living facility or a nursing home. 9.3% of patients were living in a family or friend's home, 4.7% were living at home with formal assistance, and 2.3% came to the hospital from a skilled nursing/medical facility.

#### 4.3.2. Posthospitalization

The greatest percentage of patients in the sample (32.6%) was comprised of those who were discharged to home, either with or without family support (see Table 2). Several patients did not return home after hospitalization, as indicated by a drop of approximately 12% from intake status. Of those who initially came from their homes, two were discharged to home with formal assistance, one went to a friend's home, one was discharged to an assisted living facility, and one went to a nursing home. Almost 40% of the sample was discharged to either an assisted living facility (20.9%) or nursing home (18.6%), not significantly changed from intake status.

## 5. Discussion

This study used a retrospective, chart review design to examine intake and discharge characteristics of PD patients who required inpatient psychiatric hospitalization. To our knowledge, this is the only study examining PD patients who have been psychiatrically hospitalized. Although this study examined a specific sample of patients hospitalized at an inpatient facility, results were consistent with past studies showing the high prevalence of nonmotor diagnoses in PD.

Several important observations were generated. Although PD preferentially affects men, more women were hospitalized than men. Our sample was small, so this may have been observed due to chance alone or may represent a bias of men refusing admission to a psychiatric hospital. Alternatively, male caregiver-spouses may have lower thresholds for seeking the assistance of an inpatient facility. We also found an unexpectedly high frequency of substance abuse, not generally recognized as a problem in PD. Potentially discordant admitting diagnoses of PD and DAT may prompt inpatient clinicians to reconsider the etiology of cognitive impairment in the setting of PD, as evidenced by five of 12 patients admitted with DAT having this diagnosis changed prior to discharge. Anxiety was as common a cause for hospitalization as psychosis, an indication that anxiety is underrecognized as a potentially severe problem in PD. Almost 10% of the patients were discharged to a skilled nursing or medical facility. Length of stay averaged 17.63 days, generally consistent with the length of stay for the geriatric unit at the same hospital (average length of stay 14.55 days). This length of stay suggests an average cost of over $12,000 per psychiatric hospitalization (based upon cost for community health inpatient mental health hospitalizations) [27]. Consequently, this indicates that more aggressive outpatient management might result in major cost savings, as well as reduced institutionalization in long-term care facilities.

This study had several limitations. As this is a retrospective study, extensive information was unavailable on these patients. Standardized diagnostic assessments were not carried out on the majority of patients. Comorbid diagnoses may have been missed during the initial intake interview, and PD dementia is not an optional category during intake for initial diagnosis; rather, patients would have been categorized under dementia not otherwise specified. Additionally, neurological assessment of the type and severity of Parkinson's symptoms was not carried out (or was unavailable for review). Similarly, neuropsychological testing was not routinely done to distinguish PD-related cognitive impairment from DAT.

Future research should include a prospective study examining reasons for psychiatric hospitalization. In addition, detailed analysis of the relationship between neuropsychiatric symptoms and length of stay and recurrence of hospitalizations will be beneficial. The long-term goal of this area of study would be to reduce neuropsychiatric symptoms and improve quality of life in order to reduce inpatient hospital stays. This would be carried out by an in-depth screening of neuropsychiatric symptoms, likely within a primary care or neurology office, in order to provide early treatment.

## Figures and Tables

**Table 1:** Descriptive data for inpatient sample.

Diagnosis	Intake and discharge diagnoses by DSM-IV category		Age of patients in sample by diagnosis		Length of stay (in days) by diagnosis	
	Percentage with disorder (based on intake diagnosis)	Percentage with disorder (based on discharge diagnosis)	Age (based on intake diagnosis) M (SD)	Age (based on discharge diagnosis) M (SD)	Length of stay (based on intake diagnosis) M (SD)	Length of stay (based on discharge diagnosis) M (SD)
Substance-related	*N* = 6 14.0%	*N* = 7 16.3%	*N* = 6 60.33 (14.21)	*N* = 7 62.43 (14.59)	*N* = 6 24.33 (34.65)	*N* = 7 24.57 (31.63)
Psychotic	*N* = 12 27.9%	*N* = 10 23.3%	*N* = 12 73.17 (6.03)	*N* = 10 72.10 (5.72)	*N* = 12 23.67 (29.91)	*N* = 10 20.00 (27.33)
Dementia	*N* = 21 48.8%	*N* = 21 48.8%	*N* = 21 79.14 (9.62)	*N* = 21 79.42 (9.38)	*N* = 21 19.90 (16.94)	*N* = 21 21.24 (16.95)
Mood	*N* = 23 53.5%	*N* = 19 44.2%	*N* = 23 71.30 (13.09)	*N* = 19 69.95 (14.17)	*N* = 23 13.96 (10.23)	*N* = 19 13.37 (9.34)
Anxiety	*N* = 7 16.3%	*N* = 9 20.9%	*N* = 7 71.29 (18.72)	*N* = 9 78.67 (8.76)	*N* = 7 13.71 (8.50)	*N* = 9 12.44 (8.41)
Adjustment	*N* = 1 2.3%	*N* = 2 4.7%	*N* = 1 61.00 (0.00)	*N* = 2 49.00 (16.97)	*N* = 1 4	*N* = 2 5.50 (2.12)

**Table 2:** Intake and discharge living situations.

Living situation	Intake	Discharge
Home	41.9%	34.9%
Home with formal assistance	4.7%	9.3%
Living in family or friend's home	9.3%	4.7%
Assisted living	20.9%	20.9%
Nursing home	20.9%	18.6%
Skilled nursing/medical facility	2.3%	9.3%
Unknown		2.3%
